# The Role of Magnesium in Pregnancy and in Fetal Programming of Adult Diseases

**DOI:** 10.1007/s12011-020-02513-0

**Published:** 2020-12-14

**Authors:** Daniela Fanni, C. Gerosa, V. M. Nurchi, M. Manchia, L. Saba, F. Coghe, G. Crisponi, Y. Gibo, P. Van Eyken, V. Fanos, G. Faa

**Affiliations:** 1 UOC Sezione di Anatomia patologica, Dipartimento di scienze Mediche e Sanità pubblica, University of Cagliari, Via ospedale, 54, 09124 Cagliari, Italy; 2 UOC Anatomia Patologica, AOU Cagliari, University of Cagliari, Cagliari, Italy; 3 Dipartimento di Scienze della Vita e dell’Ambiente, University of Cagliari, Cagliari, Italy; 4 UOC di Psichiatria, University of Cagliari, Cagliari, Italy; 5 UOC Radiologia, AOU Cagliari, University of Cagliari, Cagliari, Italy; 6 UOC Laboratorio Analisi, University of Cagliari, AOU Cagliari, Cagliari, Italy; 7 Hepatology Clinic, Matsumoto, Japan; 8 Department of Pathology, UZ Genk Regional Hospital, Genk, Belgium; 9 UOC Terapia Intensiva Neonatale, AOU Cagliari, University of Cagliari, Cagliari, Italy; 10 Adjunct Professor Temple University, Philadelphia, PA USA

**Keywords:** Magnesium, Trace element, Metal, Pregnancy, Fetal, Programming

## Abstract

Magnesium is an essential trace metal and a necessary factor for multiple biochemical functions in humans. Its role in biology is fundamental in over 600 enzymatic reactions implicated in protein synthesis, mitochondrial functions, neuromuscular activity, bone formation, and immune system competence. Magnesium status is relevant in fetal development during gestation and in the newborn growth during the perinatal period. Moreover, magnesium is able to influence fetal programming and disease presentation in childhood or adulthood. The aim of this review is to focus on this metal homeostasis, analyzing its normal values, the causes of hypomagnesemia, the interaction with drugs and other conditions, and the diseases associated with magnesium value alteration during pregnancy, in order to study its role in fetal programming of adult diseases. The data here reported clearly indicated the existence of a connection between magnesium status and human pathology starting from intrauterine life and extending into childhood and adulthood.

## Introduction

Magnesium is one of the ten essential metals in humans, the fourth most abundant cation, after calcium, potassium, and sodium, and the second most prevalent intracellular cation in human tissues [1]. Magnesium is a multivalent cation, pivotal for many biochemical and physiological processes, being involved in many biologic and cellular functions including protein synthesis and nucleotide metabolism [2]. Nowadays, recognition of the main role of magnesium has been progressively enlarged, thanks to its exceptional biochemical activity and, it is now recognized as an essential factor for multiple biochemical functions in human cells. Intracellular magnesium ions can bind to the cell membrane, the nucleus, and ribosomes. Magnesium is indispensable for the aggregation of ribosomes into polysomes, and so plays a key role in protein synthesis. Moreover, magnesium ions also act as cofactors for ribonucleic acid enzymes deputed specifically to recognize and to cleave the target mRNA. Magnesium is involved in the regulation of mitochondrial functions, including ATP production [3]. The role of magnesium in biology is essential: magnesium ions play a relevant role in over 600 enzymatic reactions, including energy metabolism, synthesis of fatty acids and proteins, neuromuscular excitability, and transmission of nerve impulses [4]. Mitochondria have been shown as major intracellular magnesium stores [5]. Magnesium is crucial for the formation of bone, favoring calcium assimilation into bones and contributing to the activation of vitamin D in the kidneys [6]. The highest stores of magnesium have been found in bones, followed by muscles, soft tissues, and body fluids. In clinical practice, the serum magnesium concentration is the laboratory test widely utilized to assess magnesium status, even though only about 1% of the total body magnesium is present in blood [7].

In order to be biologically active, adenosine triphosphate (ATP) requires to be bound to magnesium [8]. Moreover, magnesium performs an essential function in the transition state of ATP synthesis from ADP and inorganic phosphate. In immunological competence, magnesium ions are important, especially for the role played by Mg^2+^ in the activation of the immune system. In particular, Mg^2+^ is a cofactor of several metabolic enzymes, which are upregulated in activated immune cells [9].

The aim of this review is to focus on the importance of magnesium status in fetal development during gestation, in newborn growth in the perinatal period, and in fetal programming of diseases presenting in childhood or adulthood.

## Magnesium Homeostasis

The total Mg content of a 70-kg adult with 20% of fat is about 24 g [10]. Magnesium homeostasis is regulated by the activity of multiple molecules in the kidney and in the gut, which are important in the homeostasis of this metal. MAGT1 is a magnesium transporter localized in the endoplasmic reticulum, which transports magnesium ions to the plasma membrane of multiple cells such as T lymphocytes including CD8+ T cells that recognize microbiologic agents in order to prevent infections and CD4+ T cells that assist the activity of B cells [11]. About 99% of magnesium is stored in bones as a constituent of hydroxyapatite, skeletal muscles, and soft tissues [7, 12]. Bones’ stores of magnesium provide an exchangeable pool useful for buffering changes in the serum magnesium levels [13]. In blood, magnesium is also present inside the erythrocytes, and their magnesium concentration is considered an ideal indicator of body magnesium stores [14]. Renal regulation of these ions occurs through glomerular filtration and tubular reabsorption and is an important determinant of plasma ion concentration [15]. During the last years, it has been noticed that the tight junction proteins claudin-10 and claudin-16 have a lead role in the paracellular reabsorption of magnesium along the ascending limb of the Henle’s loop [16]. Genetic studies in patients with primary hypomagnesemia have shown “transient receptor potential melastatin 6” (TRPM6) as the principal component involved in epithelial magnesium reabsorption [17].

In experimental animal model, mutations in claudin-16 results in hypomagnesemia, while absence of claudin-10 results in hypermagnesemia. Studies carried out on newly generated mouse models provided new data on the role of the ion channel TRPM6 in the regulation of Mg^2+^ balance during prenatal development. TRPM6 expression was detected in the placenta and yolk sac, where it is essential for embryonic development [18].

These data show that TRPM6 is a determining factor for prenatal development and for maintenance of magnesium balance and survival to adulthood. Later studies confirmed that the epithelial Mg channel TRPM6 should be considered the magnesium ions’ entry pathway in the ascending limb of Henle’s loop and in the distal convoluted tubule of the kidney, where TRPM6 functions as a gatekeeper for controlling the body’s magnesium balance [19].

When the body’s stores of magnesium decline, gastrointestinal absorption, bone resorption, and renal tubular reabsorption increase, in order to normalize magnesium stores. TRPM6 was recently identified and shown to operate in active epithelial magnesium transport also in the intestine. It has been observe that in mice, dietary magnesium restriction resulted in increased absorption of magnesium ions, which was correlated with increased TRPM6 expression in kidney and in gut cells [20]. On the contrary, differently  from other trace elements, such as copper [21, 22], iron [23, 24], and aluminum [25, 26], where metal overload have severe clinical consequences, hypermagnesemia is a rare clinical occurrence, restricted to patients with impaired renal function or to newborns following administration of sulfate magnesium during pregnancy [27].

## Genetic Causes of Hypomagnesemia

Hypomagnesemia is defined as serum magnesium levels lower than 1.8 mg/dL (< 0.74 mmol/L) [28]. Hypomagnesemia is the most common disturbance of magnesium homeostasis, with a prevalence of up to 15% in the general population [29]. In 2001, Schimatschek et al. reported the prevalence of hypomagnesemia in an unselected German population of 16,000 individuals. Based on Mg lower limit of less than 0.76 mmol Mg/L, the frequency of hypomagnesemia was present in about 14.5% with higher frequencies in females and outpatients; in 33%, serum magnesium levels were lower and suboptimal [30]. The identification of molecular defects related to multiple inherited magnesium losing disorders has contributed to a better knowledge of the processes at the basis of renal magnesium handling or losing [31].

For multiple relevant physiological processes, blood magnesium levels are decisive. Therefore, persistent low magnesium stores, evidenced in clinical practice by low magnesium serum levels (hypomagnesemia), are associated with serious health risks and with the pathogenesis of multiple diseases, including cardiac arrhythmias, type 2 diabetes mellitus, osteoporosis, asthma, cardiovascular diseases, neuromuscular irritability and seizures. The recent literature has expanded significantly our knowledge on the molecular pathways involved in hypomagnesemia, thanks to the identification of novel genes and signaling pathways involved in hypomagnesemic disorders. Over a dozen genes directly or indirectly implicated in transport of magnesium ions have been identified so far and classified into four groups: (i) hypercalciuric hypomagnesemias CLDN16, CLDN19, CASR, and CLCNKB; (ii) Gitelman-like hypomagnesemias, CLCNKB, SLC12A3, BSND, KCNJ10, FYXD2, HNF1B, and PCBD1; (iii) mitochondrial hypomagnesemias, SARS2 and MT-TI; and (iv) other hypomagnesemias, TRPM6, CNMM2, EGF, EGFR, KCNA1, and FAM111A [32]. The gene CLDN16 encodes for paracellin 1 (claudin-16), a tight junction protein, which is expressed in the ascending loop of Henle and in the distal convoluted tubule, where it regulates reabsorption of magnesium [33]. Multiple mutations in the gene coding for the tight junctions claudin-16 and claudin-19 have been reported to be responsible for familial hypomagnesemia with hypercalciuria and nephrocalcinosis (FHHNC), a rare tubular disorder characterized by the dysregulation of the paracellular ion permeability of epithelia [34]. Magnesium deficiency is associated with reduced protein synthesis, serum antibody titer, and decrease immune response [35].

## Recommended Daily Allowance of Magnesium

The recommended daily allowance (RDA) is the amount of a given substance necessary for the health of human beings. The RDA for magnesium is variable with gender and age. The National Institutes of Health recommends a RDA for magnesium of 80 mg/day for children aged 1–3 years, 130 mg/day for children aged 4–8 years, and 240 mg/day for children aged 9–13 years, irrespective from gender.

The RDA values for men and women diverge after this age, and men normally require more Mg^2+^ than women, depending on their larger body mass. Furthermore, it has to be pointed out that pregnancy and lactation dictate an about 10% higher supply of magnesium, i.e., an RDA of 350–400 mg/day is recommended in pregnancy and of 310–360 mg/day during lactation, compared with 300–310 mg/day for non-pregnant or non-lactating women. Considering the 33% suboptimal serum Mg found in the German population [30], whether or not these amounts are really adequate should be verified. During pregnancy, the suggested EAR is an addition of 40 mg (1.6 mmol)/day of the RDA independently of the future mother’s age. The addition calculated for pregnancy was less than the addition in accordance with the gain in weight. However, whether implementation of magnesium intakes during pregnancy should be suggested was difficult to determine. Moreover, no data about adequate magnesium storage and increased intestinal absorption during pregnancy are available [36].

Further data regarding dietary reference values for magnesium for children in European Countries [37] maybe found in EFSA Panel on Dietetic Products, Nutrition and Allergies (NDA) Table 1.

**Table 1:** RDA values recommended for magnesium (mg/day)

Age	Male	Female	Pregnancy	Lactation
Birth to 6 months	30	30		
7–12 months	75	75		
1–3 years	80	80		
4–8 years	130	130		
9–13 years	240	240		
14–18 years	410	360	400	360
19–30 years	400	310	350	310
31–50 years	420	320	360	320
51+ years	420	320		

Source: https://ods.od.nih.gov/factsheets/Magnesium-HealthProfessional/

## Methods for Defining Magnesium Status in Clinical Practice

Various methodologies are used for assessing magnesium status. In clinical practice, serum magnesium levels are considered a Mg marker although it is not widely accepted as truly representative of Mg stores of the whole body as only < 1% of total body Mg resides in the serum (about 1% in blood). The estimation of the mean normal magnesium value in a normal person without any clinical symptom is about 0.9 mmol/L Mg. Clinically relevant symptoms were reported when serum values were less than 0.8 mmol/L Mg [38]. Other authors reported that serum magnesium levels < 1.8 mg/dL (< 0.74 mmol/L) determined hypomagnesemia [28]. Mg deficiency has been described as common but underdetected since Mg has an intracellular role and it is located not only in the plasma. Few data regarding magnesium serum levels during pregnancy are available in the scientific literature. Further studies are needed in order to reliably establish Mg status in pregnancy. Additionally, the clinical appearance of magnesium deficiency is characterized by a high variability. Moreover, the development of pathologic findings may follow long periods with marginal deficiencies. Consequently, the prevalence and the significance of magnesium deficiency is insufficiently considered and, often, it is underdiagnosed [38]. Furthermore, being magnesium primarily an intracellular cation, serum magnesium levels should be always considered as an insufficient predictor of the real magnesium content in the whole body. Serum magnesium levels < 2.0 mg/dL, with a urinary excretion of 40–80 mg/day, are highly indicative of magnesium deficiency [39]. Patients affected by celiac disease (CD) are susceptible to suffer hypomagnesemia. CD-dependent enteropathy causes numerous nutritional deficiencies, which involve both macro- and micronutrients, including magnesium [40].

## Mg Interactions with Proton Pump Inhibitor Medications

In recent years, some drugs have been associated with magnesium deficiency. A large experimental evidence links the chronic use of PPIs with hypomagnesemia, presumably due to decreased intestinal absorption and consequent systemic magnesium deficiency [41]. In patients under chronic therapy with PPIs, magnesium deficiency has been hypothesized as the cause of life-threatening problems, the principal ones being secondary electrolyte disturbances [42]. Experimental studies in mice evidenced that maternal Mg deficiency can result in behavioral deficits in the offspring during adult life, confirming a major role for an optimal magnesium status during pregnancy on the health status of newborns extending to adulthood [43]. Contrasting data regarding the safety of PPIs for long-term use have been published. In the last few years, the association between the chronic use of PPIs and hypomagnesemia has been questioned. In a study on a large cohort of chronic PPI users, no statistical difference in magnesium serum levels between patients on PPI therapy and control subjects was found. Analogously, no significant differences between subjects in cure with PPIs at variable doses, with or without associated diuretics, and the control group were observed. On this base, previous studies on hypomagnesemia and PPI use should be reconsidered with caution [44]. No data are available at the best of our knowledge, regarding magnesium status in pregnant women undergoing PPI use.

## Foods Rich in Magnesium

A normal diet can provide adequate magnesium, but often does not. Dietary surveys of people from Europe and the USA evidenced that daily intake of magnesium is lower than the recommended dosage [45]. This is likely due to modern processing, especially of grains, oilseeds, and sugar crops, which removes much of the naturally occurring magnesium [46] (http://magnesiumeducation.com/whole-vs-refined-food). In fact, many foods contain magnesium, including nuts, unrefined grains and grain products, fish, seafood, vegetables, legumes, berries, banana coffee, and cocoa drinks. Even tap and bottled water can make a significant contribution to magnesium intake [37].

### The Role of Magnesium During Gestation and in Fetal Programming of Adult Diseases

Chronic primary magnesium deficiency is frequent, about 20% of women are receiving low intakes of magnesium and consuming less than two-thirds of the RDA. Pregnant women are at higher risk of developing magnesium deficiency, with maternal, fetal, and pediatric consequences. A magnesium deficiency status during gestation may interfere with fetal growth and development and may favor premature labor. Preterm delivery is due to uterine hyperexcitability caused by chronic maternal Mg deficiency and is intensified in situations of maternal stress. Gestational magnesium deficiency may induce major maternal, fetal, and pediatric effects which might last throughout life [47].

Consequences of maternal primary Mg deficiency are not restricted to the prenatal and to the perinatal period. Low magnesium levels during pregnancy may have important consequences throughout life, with hypomagnesemia representing an important factor in the broad spectrum of the fetal programming theory of diseases presenting later in life, in childhood, or in adulthood [48]. In rats, maternal restriction of magnesium in the diet during gestation resulted in the increase in body fat, induced insulin resistance, and impaired glucose tolerance in pups by 6 months of age [42]. In mice, maternal hypomagnesemia was able to program a behavior characterized by anxiety in the offspring, which was maintained in adulthood [43]. In humans, maternal hypomagnesemia has also been associated with the insurgence of the metabolic syndrome later in life [49]. Moreover, inadequate maternal intake of magnesium during pregnancy is at the basis of fetal hypomagnesemia that has been associated with restricted fetal growth. Many epidemiological studies have associated intrauterine growth restriction (IUGR) with an increased risk of undergoing insulin resistance later in life, suggesting that chronic intrauterine magnesium deficiency might result in IUGR. According to this hypothesis, severe magnesium deficiency in pregnant women might program the insurgence of insulin resistance in newborns, with important consequences after birth, ending with the insurgence of metabolic syndrome in childhood or adulthood [50].

Gender differences were reported regarding the influence of maternal hypomagnesemia on the susceptibility of the offspring to develop renal and cardiovascular diseases later in life. In a mouse model, low magnesium levels in the maternal diet during gestation resulted in increased urine flow both in males and females, whereas a reduction in renal excretion of magnesium was restricted to males. Both male and female offspring from magnesium-deficient dams showed an increased in urine flow at 6 months of age, in the absence of significant differences in water consumption. These changes in urinary volume could affect the magnesium requirements in male, but not female, offspring of magnesium deficiency dams later in life [51].

A diagnosis of IUGR, often associated with preterm birth, is associated with increased risk in the affected newborns of adverse perinatal and neurodevelopmental outcomes presenting from childhood to adulthood. The prevention of the risk to these adverse outcomes in neonates with IUGR, included in the spectrum of fetal programming of multiple human diseases, remains an unresolved dilemma [52]. Regarding the hypothesis that consequences of magnesium deficiency during gestation might last in offspring throughout life, small for gestational age (SGA) infants have been reported to have an increased risk of insulin resistance in adult life. Moreover, intrauterine magnesium deficiency in the fetus may program insulin resistance after birth and may induce the insurgence of the metabolic syndrome later in life [53].

In recent years, the administration of magnesium sulfate during gestation has been introduced in clinical trials aimed to obtain neuroprotection in small-for-gestational-age babies or when preterm delivery is programmed. Magnesium sulfate has been shown to readily cross the vasculosincytial membrane in the terminal villi of the placenta being transferred from maternal to fetal circulation within an hour of administration. The maternal administration of magnesium sulfate during the perinatal period has been demonstrated to exert neuroprotective effects on the fetus, probably through the following three major mechanisms: (i) reduction of intracellular calcium entry; (ii) block of glutamate and other neurotransmitter receptors responsible for neuronal cell death; and (iii) modulation of the actions of oxygen-free radicals and proinflammatory cytokines [54]. The risk of adult-onset diseases such as insulin resistance and metabolic syndrome has been reported to be higher in children with a low birth weight and whose mother was malnourished during pregnancy. At experimental level, the offspring of pregnant rats fed a low magnesium diet, showed hypermethylation in the hepatic 11β-hydroxysteroid dehydrogenase-2 promoter [49]. These data clearly indicate that magnesium deficiency during gestation may cause the epigenetic dysregulation of gene expression in the fetus, promoting different metabolic phenotypes in the newborn that might persist throughout life. Very recently, the maternal magnesium status during gestation has been correlated with the cognitive outcomes of the neonates. To this end, magnesium serum levels were measured at 26–28 weeks of gestation and the cognitive development of the infants was evaluated at 4 years of age. Higher maternal magnesium serum levels were associated with higher scores in letters and writing identification, suggesting the existence of long-term influences of maternal magnesium on child’s cognitive development [55].

### Magnesium and Intrauterine Growth Restriction

Trace elements, including magnesium, are an essential nutritional component for humans, particularly in the intrauterine life when fetal development occurs [56], and inadequate tissue concentrations of magnesium may have significant adverse effects on fetal weight at birth [52]. The need for magnesium increases during pregnancy, but the majority of pregnant women do not meet this increased need. Hypomagnesemia is frequently observed in pregnant women, both in developing and developed countries. Magnesium deficiency during pregnancy is associated with a higher health risk for both mother and newborn, including restricted fetal growth, intrauterine growth restriction, gestational diabetes, preterm labor, and pre-eclampsia. Low magnesium intakes during pregnancy may also have relevant consequences on the health status throughout life, such as the insurgence of metabolic syndrome later in life. In order to prevent all these adverse effects, pregnant women should be counseled to increase their intake of magnesium-rich foods, such as beans, nuts, seeds, leafy greens, or supplements of magnesium in the diet. According to these data, magnesium may enter as a main actor in the “fetal origin” hypothesis of multiple human diseases, including the predisposition to develop a metabolic syndrome in childhood or adulthood [52, 57].

Studies of gestational Mg^2+^ deficiency in animals confirmed that it may have adverse effects on the mode of birth and on postnatal life. Maternal hypomagnesemia is associated with IUGR and premature labor, by inducing uterine hyperexcitability and overactivity, ending with abortion or preterm birth. The hypothesis that magnesium deficiency might be the basis of hyperreactivity of uterine muscle cells led to suggest magnesium supplementation during pregnancy as an optimal adjuvant therapy for the prevention of prematurity [47].

In a study focused on the possible influence of supplements on pregnancy outcome, in magnesium-supplemented pregnant mothers, the risk for neonates to undergo a low birth weight was significantly lower, as compared with mothers with no magnesium supplementation [58].

The hypothesis that magnesium deficiency might be at the basis of the insurgence of IUGR has been confirmed in a study aimed to correlate magnesium levels in umbilical vein and in maternal peripheral blood with fetal weight at birth [59]. In this study, newborns of pregnant women supplemented with magnesium sulfate (25% magnesium sulfate 20 ml in 5% glucose 500 ml i.v.) were characterized by a birth weight significantly higher than that of unsupplemented women with low serum magnesium levels. All these data taken together to strongly support recommendations for higher magnesium during pregnancy are mandatory.

### Magnesium and Preterm Labor

A possible influence of maternal magnesium status on the risk of preterm labor has been reported by many groups in recent years.

Two meta-analyses based on multiple studies related to the efficacy of magnesium sulfate supplementation in women in threatened preterm labor evidenced no real help of magnesium sulfate therapy for preterm labor [60, 61]. A breakthrough in our knowledge on the value of magnesium sulfate tocolytic therapy is represented by two studies of Doyle et al. on antenatal magnesium sulfate for women at risk for preterm birth and neurologic outcome in preterm infants. These studies underlined the usefulness of magnesium sulfate therapy in mothers in danger of preterm labor, demonstrating that antenatal magnesium sulfate administration is associated with better outcomes among the infants from such pregnancies, even though it does not alleviate the preterm labor [62, 63].

#### Magnesium, Pre-eclampsia, and Eclampsia

It has been claimed that magnesium supplementation of pregnant women might reduce the risk of IUGR of the fetus, increase birthweight, and reduce by half the incidence of eclampsia [64]. Pre-eclampsia, which can cause damage to liver and kidneys, is a syndrome occurring in pregnant women, characterized by high maternal blood pressure and raised albumin levels in urine. The hypothesis that pre-eclampsia might be prevented from progressing to eclampsia with magnesium sulfate was the subject of an international randomized controlled clinical trial, named MagPie, carried out on 10,000 women [64].

Magnesium sulfate administered to women with pre-eclampsia more than halves the risk of eclampsia, probably reducing the risk of maternal death during perinatal period. No essential detrimental side effects were apparent in the short term, for either mother or baby. Maternal treatment with magnesium sulfate was not associated with a sharp difference in the risk of death or disability for children after 18 months [65]. Moreover, the reduction in the risk of eclampsia following magnesium sulfate administration was not associated with an increase of death or disability in women after 2 years [66]. The ability of magnesium sulfate treatment to reduce more than 50% the risk of eclampsia (among pre-eclamptic women) and probably maternal death has been confirmed by a meta-analysis from the Cochrane Pregnancy and Childbirth Group’s Trial Register and the Cochrane Central Register of Controlled Trial Register [67]. Additionally, magnesium sulfate for women with eclampsia has been shown to reduce the risk of maternal death and of recurrence of seizures [68, 69]. Further studies have confirmed that the use of magnesium is effective in halting the progression of pre-eclampsia to eclampsia [70]. Hypermagnesemia usually occurs in pre-eclamptic women after Mg therapy [29]. The molecular pathways, through which magnesium could act, halting the insurgence and progression of maternal hypertension, are not fully understood. Magnesium ions might act as calcium antagonists and/or as inducers of the release of prostaglandins, that mediates blood pressure [71]. A recent study proposed the use in pregnant women of individualized doses of i.v. magnesium salts, according to body weight and serum creatinine, with the aim to reach a personalized approach to magnesium use in the prevention of pre-eclampsia [72]. Conflicting results arose from a meta-analysis on oral magnesium supplementation during pregnancy. This analysis showed that oral magnesium supplementation does not appear to prevent pre-eclampsia although there were not enough high-quality trials to give an overall final conclusion except when limited to two high-quality trials [73]. Any differences have not been noticed between babies who received magnesium from their mothers during gestation and newborns born from mothers who did not receive magnesium. In particular, maternal magnesium supplementation did not reduce the incidence of fetal growth restriction, neither the incidence of pre-eclampsia in pregnant women [73]. Differences have been reported regarding administration method of magnesium in the prevention of pre-eclampsia. Intramuscular administration of magnesium sulfate was observed to be superior than phenytoin for the prevention of eclampsia in hypertensive pregnant women, i.e., those with pre-eclampsia [74]. On the other hand, oral magnesium supplementation was associated with significantly fewer babies with an Apgar score of less than 7 at 5 min (RR, 0.34; 95% CI, 0.15 to 0.80; four trials, 1083 infants), with meconium-stained liquor (RR, 0.79; 95% CI, 0.63 to 0.99; one trial, 4082 infants), late fetal heart decelerations (RR, 0.68; 95% CI, 0.53 to 0.88; one trial, 4082 infants), and mild hypoxic-ischemic encephalopathy (RR, 0.38; 95% CI, 0.15 to 0.98; one trial, 4082 infants). Moreover, women receiving oral magnesium were significantly less likely to require hospitalization during pregnancy (RR, 0.65; 95% CI, 0.48 to 0.86; three trials, 1158 women) [73].

### Magnesium and SIDS

The area of the relationship between magnesium status and sudden infant death syndrome (SIDS) is a promising one for research and at the moment provides just very sophisticated speculation. SIDS represents a leading cause of death during the first year. Many years ago, the hypothesis that SIDS might be magnesium dependent has been proposed. According to this hypothesis, low magnesium intakes to the fetus during gestation might lead to impaired control of thermoregulation mechanisms of the infant, ending with severe dysthermias [75]. Further studies from the same author suggested that prophylaxis of SIDS should include atoxic nutritional magnesium therapy for pregnant women with a total absence of light at night for the infant [47].

In the absence of any certain knowledge regarding the etiology and pathogenesis of SIDS, recent theories have proposed that infant sudden death might be a magnesium-dependent disease of the transition from chemical to physical thermoregulation. This theory leads to promote an investigation of the usefulness of magnesium supplementation for the prevention of SIDS in pregnant and lactating women, through the first year of life [76].

### Magnesium and NEC

Recent studies since the 2010 adoption of the magnesium sulfate for early preterm birth by the College of Obstetrics and Gynecology reported conflicting results on the relationship between antenatal magnesium sulfate (MgSO_4_) exposure and a higher risk of insurgence of necrotizing enterocolitis (NEC) in preterms [77]. Conflicting results have been reported on the relationship between antenatal magnesium sulfate exposure and neonatal intestinal injury because the vast majority of studies did not assess magnesium sulfate exposure quantitatively and none reported the exposure timing. In order to assess whether there is any relationship between antenatal magnesium dose and time of administration of therapy with intestinal injury in extreme preterms, 302 neonates born prematurely or with birth weight < 1000 g, whose mothers underwent magnesium sulfate supplementation during the last week before birth were followed-up. Antenatal magnesium sulfate exposure in extreme preterm newborns was not associated with an increased risk of necrotizing enterocolitis or of other intestinal injuries. On the contrary, necrotizing enterocolitis and spontaneous intestinal perforation were both decreased in the neonates of magnesium-supplemented mothers, in a dose-dependent manner, indicating a possible protective effect against NEC of magnesium maternal supplementation. Each 10 g increase in magnesium sulfate cumulative dose correlated with decrease in NEC death by 18.9% prior to discharge and by 21.9% during the first 2 weeks of life [78].

### Magnesium and Fetal Neuroprotection

The World Health Organization (WHO), in its 2015 recommendations on interventions to improve preterm birth outcomes, strongly recommends the use of magnesium sulfate for fetal neuroprotection. The WHO recommendation has been followed by a meta-analysis aimed at providing guidelines for the antenatal administration of magnesium sulfate for fetal neuroprotection of preterm infants [79]. In this study, magnesium sulfate for fetal neuroprotection should be considered only in women presenting with imminent preterm birth at < 30 weeks of gestation. According to the multiple studies taken in consideration with this meta-analysis, a starting loading dose of 4–6 g of magnesium sulfate administered intravenously was suggested followed by a maintenance dose of 1–3 g. Differences were found regarding the time of administration ranging from bolus or 10–30 min. Antenatal magnesium sulfate administration reduced the risk of death or cerebral palsy and the inability to walk without assistance at 2 years of age [79].

### Magnesium and Leg Cramps

Nocturnal leg cramps (NLCs) are a common symptom in the general population, often associated with sleep disturbance. The possible mechanisms for the use of magnesium supplementation in subjects affected by leg cramps might be related to numerous processes that affect muscle function including oxygen uptake, energy production, and electrolyte balance [80]. The possible therapeutic effects of magnesium supplementation on NLCs have been discussed in a meta-analysis. In this study, the data of multiple randomized controlled trials, comparing the efficacy of magnesium in the therapy of nocturnal leg cramps with other treatments, were analyzed [81]. The conclusions of this meta-analysis were in favor of a mild effect of magnesium supplementation in the therapy of leg cramps in pregnant women, in the absence of significant efficacy in the general population. On the basis of these data, a further study confirmed the utility of magnesium in the treatment of leg cramps occurring in women during gestation. Oral administration of magnesium lowered the frequency and intensity of leg cramps in pregnant women, clearly indicating magnesium supplementation as a treatment of choice for pregnancy-induced leg cramps [82]. Conflicting results on the efficacy of magnesium supplementation in the therapy of leg cramps in pregnant women were reported in another meta-analysis [83], in which magnesium, taken orally by pregnant mothers for 2 to 4 weeks, did not significantly reduce the frequency and intensity of leg cramps compared with placebo or no treatment. Caution should be taken, according to the authors of this meta-analysis on the interpretation of data regarding the efficacy of magnesium in treating leg cramps. It is unclear from the evidence reviewed whether oral magnesium might provide an effective treatment for leg cramps, due to poor study design and trials being too small to address the question reasonably. Moreover, no magnesium dose is mentioned at all in the discussion of varying results.

### Magnesium and Asthma in Children

In recent years, magnesium has been introduced as a new actor in the therapy of asthma attacks. As a consequence, the relationship between magnesium status and asthma is considered an area of promising research. Based on the knowledge that magnesium ions may induce vascular relaxation through their influence on calcium channel activity in smooth muscle cells of the arterial wall, it has been hypothesized that Mg^2+^ might have the same effect on muscle cells of the small airways. This hypothesis was verified in multiple clinical trials carried out in subjects with acute severe asthma admitted to emergency room. The results of these studies lay stress on the beneficial effects of therapies based on intravenous administration of magnesium in patients presenting with severe acute asthma [84]. The usefulness of intravenous administration of magnesium sulfate in children affected by asthma has been confirmed by a recent meta-analysis, which also evidenced that the use of nebulized magnesium sulfate has no significant effects on respiratory function [85].

## Conclusions

**Fig. 1:**
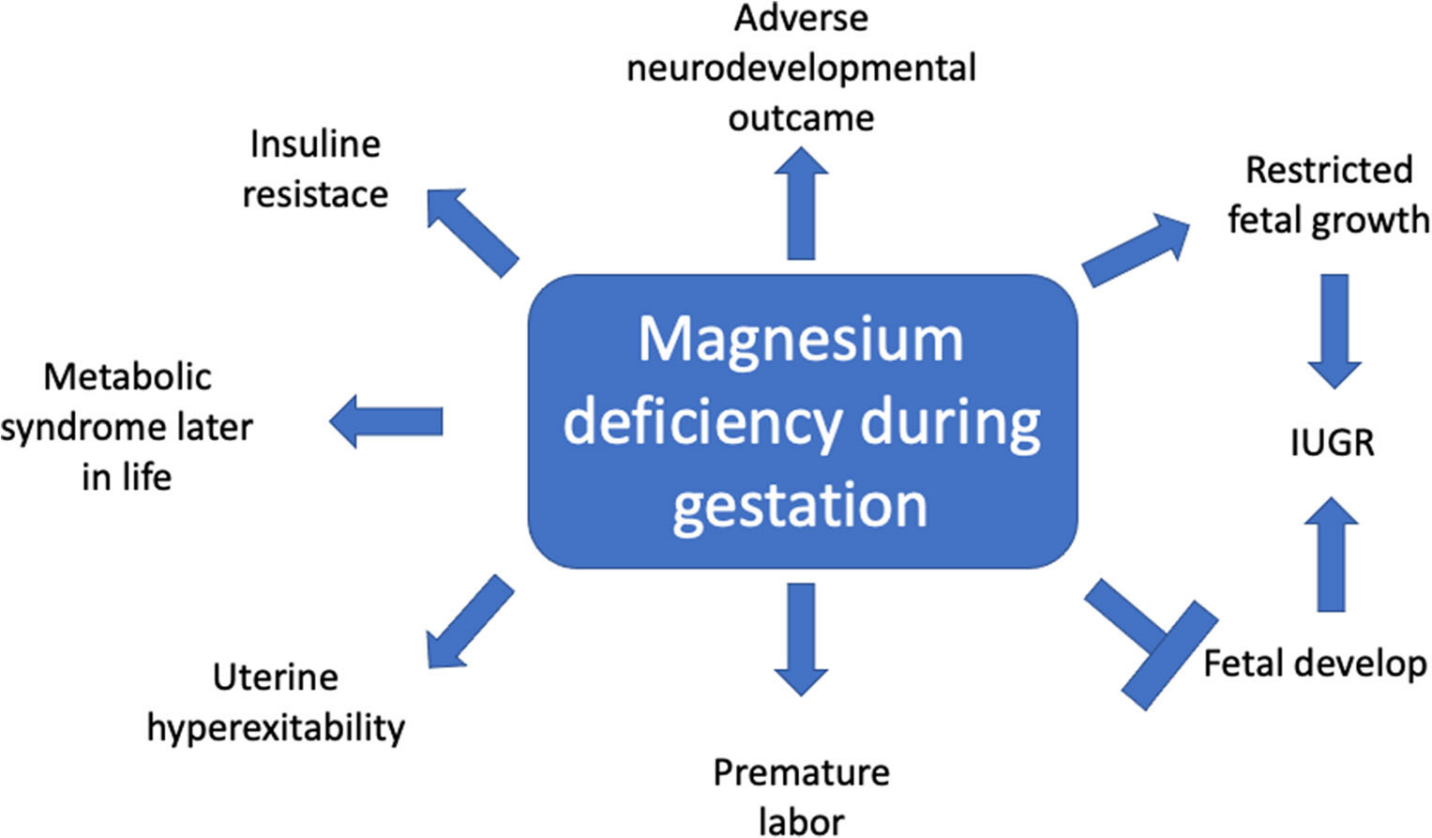
Main consequences to magnesium deficiency during gestation

In the era of the sartorial and individualized medicine, crucial efforts have been made in recent years to identify the role of trace elements in pathology and clinical practice. This review focuses especially on the role of maternal magnesium stores during gestation, as a key factor in prenatal fetal development and in postnatal life. In this review, we reported multiple evidences that support this hypothesis at the *current* state of the research. In these, we want to underline the crucial role of TRPM6, the principal factor involved in magnesium reabsorption, in fetal development and postnatal life [18]; the ability of gestational magnesium deficiency to interfere with the fetal growth and development [47]; and the consequences of maternal magnesium deficiency during pregnancy on health status at birth in childhood and adulthood [49] (Fig. 1). Pregnant women are exposed to the risk of developing magnesium deficiency, ending with major changes in fetal growth and with higher incidence of preterm labor. Consequences of maternal magnesium deficiency during pregnancy may have important consequences on the status of the newborn throughout life, as suggested before [86]. According to the data reported here, low maternal magnesium stores during gestation should be included among the multiple factors at the basis of the fetal programming of adult disease. To conclude, the scenario regarding the role of magnesium in physiology and human pathology is developing, leading to improvement of our knowledge on the consequences of inadequate intake, starting from the intrauterine life.
